# MEGADOCK-Web: an integrated database of high-throughput structure-based protein-protein interaction predictions

**DOI:** 10.1186/s12859-018-2073-x

**Published:** 2018-05-08

**Authors:** Takanori Hayashi, Yuri Matsuzaki, Keisuke Yanagisawa, Masahito Ohue, Yutaka Akiyama

**Affiliations:** 1 Department of Computer Science, School of Computing, Tokyo Institute of Technology, 2-12-1 W8-76 Ookayama, Meguro-ku, Tokyo, 152-8550 Japan; 2 Education Academy of Computational Life Sciences, Tokyo Institute of Technology, 2-12-1 W8-93 Ookayama, Meguro-ku, Tokyo, 152-8550 Japan; 3 Advanced Computational Drug Discovery Unit (ACDD), Institute of Innovative Research, Tokyo Institute of Technology, 4259 Nagatsutacho, Midori-ku, Yokohama, Kanagawa 226-8501 Japan; 4 AIST-Tokyo Tech Real World Big-Data Computation Open Innovation Laboratory (RWBC-OIL), National Institute of Advanced Industrial Science and Technology (AIST), 1-1-1 Umezono, Tsukuba, Ibaraki 305-8560 Japan; 5 Molecular Profiling Research Center for Drug Discovery (molprof), National Institute of Advanced Industrial Science and Technology (AIST), 2-4-7 Aomi, Koto-ku, Tokyo, 135-0064 Japan

**Keywords:** Protein-protein interaction, Protein-protein docking, Predicted protein-protein interaction database, MEGADOCK, MEGADOCK-Web

## Abstract

**Background:**

Protein-protein interactions (PPIs) play several roles in living cells, and computational PPI prediction is a major focus of many researchers. The three-dimensional (3D) structure and binding surface are important for the design of PPI inhibitors. Therefore, rigid body protein-protein docking calculations for two protein structures are expected to allow elucidation of PPIs different from known complexes in terms of 3D structures because known PPI information is not explicitly required. We have developed rapid PPI prediction software based on protein-protein docking, called MEGADOCK. In order to fully utilize the benefits of computational PPI predictions, it is necessary to construct a comprehensive database to gather prediction results and their predicted 3D complex structures and to make them easily accessible. Although several databases exist that provide predicted PPIs, the previous databases do not contain a sufficient number of entries for the purpose of discovering novel PPIs.

**Results:**

In this study, we constructed an integrated database of MEGADOCK PPI predictions, named MEGADOCK-Web. MEGADOCK-Web provides more than 10 times the number of PPI predictions than previous databases and enables users to conduct PPI predictions that cannot be found in conventional PPI prediction databases. In MEGADOCK-Web, there are 7528 protein chains and 28,331,628 predicted PPIs from all possible combinations of those proteins. Each protein structure is annotated with PDB ID, chain ID, UniProt AC, related KEGG pathway IDs, and known PPI pairs. Additionally, MEGADOCK-Web provides four powerful functions: 1) searching precalculated PPI predictions, 2) providing annotations for each predicted protein pair with an experimentally known PPI, 3) visualizing candidates that may interact with the query protein on biochemical pathways, and 4) visualizing predicted complex structures through a 3D molecular viewer.

**Conclusion:**

MEGADOCK-Web provides a huge amount of comprehensive PPI predictions based on docking calculations with biochemical pathways and enables users to easily and quickly assess PPI feasibilities by archiving PPI predictions. MEGADOCK-Web also promotes the discovery of new PPIs and protein functions and is freely available for use at http://www.bi.cs.titech.ac.jp/megadock-web/.

**Electronic supplementary material:**

The online version of this article (10.1186/s12859-018-2073-x) contains supplementary material, which is available to authorized users.

## Background

Many proteins interact with each other in nature [1]; these interactions are called protein-protein interactions (PPIs). PPIs play crucial roles in living cells, including signal transduction and regulation of metabolic pathways. In recent years, PPI inhibitors and candidate compounds have also been developed [2, 3] in drug discovery to treat PPI-related diseases. Therefore, understanding of PPIs is important not only for elucidation of biological phenomena but also for pharmaceutical research. PPIs are identified by biochemical experiments, such as the yeast two-hybrid method [4], mass spectrometry [5], and cocrystallization of protein complexes [6]. Although many proteins and their functions have been discovered each year, the discovery of PPIs by biochemical assays is time-consuming and expensive. In addition, several PPIs are involved in protein signaling networks, and many attempts have been made to elucidate the exhaustive interactions among groups of proteins within a specific cellular function category through experiments and calculations [7–9]. In order to improve our understanding of biological processes, computational prediction of PPIs, which is less time-consuming and less expensive than experimental approaches, is needed to discover proteins with a high likelihood of involvement in PPIs [10, 11].

Computational PPI prediction methods can be categorized into three types: 1) those utilizing the amino acid sequence information of the protein [12–14], 2) those based on co-evolution information [15], and 3) those using the three-dimensional (3D) structure of the protein [16–22]. The third method, based on the protein 3D structure, has been shown to be promising because elucidation of the 3D structure and binding surface localization of the protein can facilitate the design of PPI inhibitors [23, 24]. Methods using the 3D structure of proteins are further divided into two types: 3-a) methods based on sequence homology and structural similarity with known complex structures [16–18], and 3-b) methods based on rigid body docking of a pair of protein structures [19–22]. Since rigid body docking for two protein structures does not require a priori PPI information, PPIs whose interfaces are not necessarily similar to known complexes in terms of 3D structures or of amino acid compositions may be found by rigid body docking. To predict such novel PPIs, we developed a structure-based PPI prediction software program named MEGADOCK [25]. MEGADOCK uses a template-free (i.e., no reference to experimentally determined cocrystal structures) and fast Fourier transform (FFT)-based rigid body protein-protein docking method. Since the evaluation function of MEGADOCK is computable at high speed, MEGADOCK is faster than other template-free docking tools; for example, MEGADOCK is ~ 10 times faster than ZDOCK [25, 26]. Additionally, its implementation of multithreading, GPU acceleration, and MPI parallelization enables 30,000 PPI predictions in 2 h with 70-node GPU-equipped computing clusters [27].

However, in order to utilize PPI predictions (including predicted 3D complex structures), it is necessary to archive prediction results and their 3D structures and make this information easily accessible. For example, some existing databases, such as PrePPI [16], HOMCOS [17], PRISM [28], and Interactome3D [29], provide PPI predictions. PrePPI provides highly confident PPI predictions by combining structural, functional, evolutionary, and expression information. HOMCOS can predict PPIs on demand using query protein homology. PRISM can predict PPIs on demand based on known template protein-protein interfaces and provides a small database that includes PPI predictions. Interactome3D provides PPI predictions within PPI networks. Unfortunately, it is difficult for such systems to identify PPIs having 3D structures that differ from those of known complexes because they are based on a known PPI template. For example, it is difficult to search the predicted complex structures of adenomatous polyposis coli (APC) protein and Axin protein, a well-known PPI pair [30, 31], using these databases. In the PrePPI database, no predicted structures of the APC and Axin complex are available because PrePPI shows only high-prediction-scored complex structures. In the case of HOMCOS, it is impossible to predict the PPI because homologous proteins consisting of heterodimers have not been reported. In PRISM, the PPI prediction between AOC and Axin is not provided because of the small size of the database. Although PRISM can build predicted complex structure models on-demand, it takes about 1 h to obtain the model. In the case of Interactome3D, a predicted complex structure model is not obtained because it provides only a part of the experimentally determined structure, even if the full-length structure of APC exists Therefore, a novel database that contains exhaustive PPI predictions from the protein structure is needed to discover PPIs that have structures different from those of known PPIs.

In this study, we developed a database containing comprehensive PPI predictions by MEGADOCK among 7528 representative structures of human protein chains registered in the Protein Data Bank (PDB) [32] (a total of 28,331,628 PPI predictions) and a web interface for the database, named MEGADOCK-Web. MEGADOCK-Web provides the following four powerful functions in order to make the predicted PPI information more useful: 1) searching PPI predictions, 2) providing annotations for each predicted protein pair with known PPIs, 3) visualizing candidates of interactions with query proteins in biochemical pathways, and 4) visualizing predicted complex structures using a web browser-based 3D molecular viewer. These functions will allow users to easily access comprehensive PPI predictions from a web browser and facilitate discovery of unknown PPIs.

## Construction and contents

**Fig. 1:**
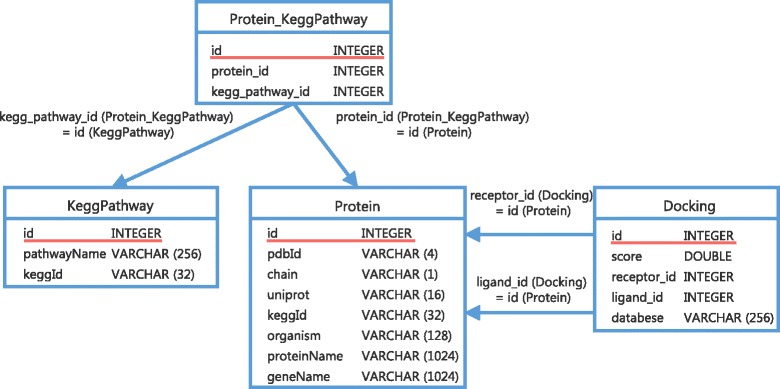
Database schema. Each protein has a PDB ID, chain ID, UniProt AC, protein name, gene name, source organism, KEGG pathways to which it belongs, and docking information. Docking describes the results of the prediction and has information on the receptor and ligand for the protein, the databases to which the protein is registered, and *PPIScore*. The red underline in the database columns indicates the primary key in each database table. Note that “column *X*1 (table *T*1) = column *X*2 (table *T*2)” for each arrow indicates that column *X*1 in database table *T*1 matches column *X*2 in database table *T*2, and these columns are the foreign key

In this section, we describe the construction of MEGADOCK-Web and the contents stored in MEGADOCK-Web. Figure 1 shows the database schema of MEGADOCK-Web. The database of MEGADOCK-Web consists of three models: 1) Protein, 2) Docking, and 3) Pathway. These attributes provide protein structure, predicted PPIs annotated with experimentally known PPI information, and biochemical pathway information in detail.

### System architecture

**Fig. 2:**
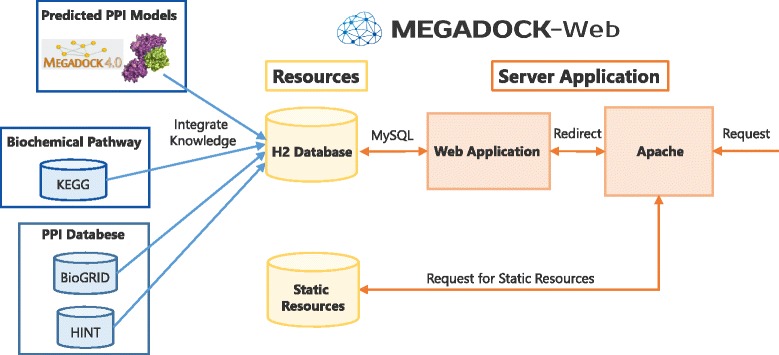
Archtecture of MEGADOCK-Web. We integrated KEGG, BioGRID, HINT, and predicted PPI information in H2 Database in advance. When a GET or POST request comes, Apache distributes the request depending on whether access to static resources or access to web pages. In the latter case, the web application returns the appropriate page referring to DB with MySQL

We developed MEGADOCK-Web using Play Framework [33], which is a Java and Scala’s web application framework including components and application programming interfaces (APIs) necessary for web application development. Figure 2 depicts the system architecture of MEGADOCK-Web. MEGADOCK-Web adopts a client-server model in the same way as a general web application. Requests from clients are classified into requests to static resources (such as protein PDB files and docking result files) and requests to the web pages. In the former case, Apache directly returns the corresponding resources to clients. In the latter case, Apache redirects the requests to the web application running on the local port. For server-side implementation, we stored information in the relational database H2 Database Engine [34] with MySQL [35] wrapped around Play Framework. Receiving requests from users invokes the web application, which generates HTML dynamically to refer to the database. The server-side implementation is multithreaded to respond immediately. Javascript and jQuery are adopted as client-side implementation tools to control web pages.

### Protein structures

This section explains the protein structures registered in MEGADOCK-Web. Since it is important for supporting drug discovery to integrate data for *Homo sapiens*, 3775 PDB entries were obtained from the PDB based on the following criteria: protein (not nucleic acids or complexes with nucleic acids); taxonomy of protein is *H. sapiens*; resolution of 2.5 Å or less; and nonredundant so that the sequence similarity is less than 90% using the PDB search function, which utilizes NCBI BLASTClust [36].

These PDB entries were divided into chain units, and we excluded those with less than 25 residues or with mutations. As a result, 7528 protein chain structures were subjected to PPI prediction. In addition, we annotated each protein chain structure with the ID of UniProt [37] (UniProt AC), the ID of Kyoto Encyclopedia of Genes and Genomes (KEGG) [38], organism name (currently only *H. sapiens*), protein name, and gene name using Bioservices [39], a package for Python.

### Known PPI pairs

MEGADOCK-Web shows known PPI pairs as well as vast number of prediction results. HINT [40] (acquired July 13, 2016) and BioGRID [41] (acquired July 13, 2016) are used as databases of known PPI pairs. MEGADOCK-Web shows known PPI information based on UniProt AC because these databases describe known PPIs based on UniProtAC.

### Biochemical pathway information

MEGADOCK-Web uses KEGG pathways as biochemical pathways in order to help understand protein functions. The KEGG pathway is a collection of manually drawn pathway maps representing knowledge on molecular interaction, reaction, and relation networks. We obtained KEGG pathway information for each protein via the web API upon startup of the server just once.

### Predicted PPI information

MEGADOCK-Web integrates exhaustive PPI predictions among 7528 protein chain structures using MEGADOCK. All-to-all PPI predictions (a total of 28,331,628) are stored in MEGADOCK-Web.

The procedure for PPI predictions by MEGADOCK is as follows. First, hydrogens and small molecules in HETATM rows in PDB files, such as cofactors and small ligands, were excluded in the predocking process. Secondly, predicted protein-protein conformations were created with MEGADOCK. MEGADOCK outputs the three best translational positions that provide the best value of docking evaluation function (*DockingScore*) per specific protein rotation, and 3600 patterns of protein rotations are computed, resulting in 10,800 predicted conformations in total. To calculate the *DockingScore* for each position, we used methods described in our previous papers [25, 42]. MEGADOCK uses FFT to enable an efficient global docking search on a 3D grid, and calculates shape complementarity, electrostatic interactions, and desolvation free energy [42]. Finally, from each *DockingScore*, MEGADOCK calculates the *PPIScore*, which describes the possibility of a PPI. *PPIScore* is defined as *PPIScore* = (*S*_*top*_ − *μ*)/*σ*, where *S*_*top*_ is the highest *DockingScore* in the protein pair, *μ* is the average of 10,800 *DockingScore*s, and *σ* is the standard deviation of these scores [24]. Higher *PPIScores* indicate a higher possibility of a PPI. Although *DockingScore* cannot be compared between different pairs of proteins, the *PPIScore*, which is normalized *DockingScore*, represents how likely the PPI is to occur. The sensitivity of PPI prediction can be adjusted by tuning the threshold value of the *PPIScore*. Additional file 1: Figure S1 illustrates the precision and recall of MEGADOCK for each threshold. We also reported the ability of MEGADOCK using real pathway datasets in our previous studies [9, 43, 44].

As a result, we calculated 7528^2^ = 56,670,784 predictions since the number of registered 3D protein chain structures was 7528. Reversing the receptor file and the ligand file generally allows predictions to be performed on the same protein pair. Notably, FFT-based docking, such as MEGADOCK, usually shows different results (*DockingScore*s) when the order of PDB input pair is switched. In order to avoid such redundancy for users, we stored the protein pairs that had higher *PPIScores* for prediction between the same PDB files. As a result, MEGADOCK-Web displays _7528_**C**_2_ + 7528 (homo dimers) = 28,331,628 PPI predictions. The total computation time was approximately 500 CPU years.

## Utility

**Fig. 3:**
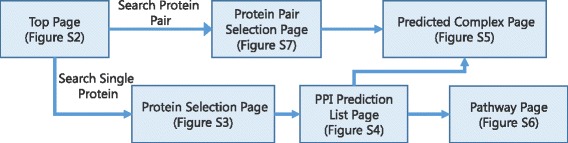
Page transition diagram of MEGADOCK-Web. For a single query, users can transit to the PPI prediction list page via the protein selection page. From this page, it is possible to transit to the pathway selection page to which the predicted binder belongs and to the prediction complex display page. For two queries, it is possible to transit to the prediction complex display page through the protein pair selection page

Figure 3 provides the page transition diagram of MEGADOCK-Web. In this section, we explain the utilities of MEGADOCK-Web in three situations: 1) searching for PPI candidates of a query protein, 2) searching for PPI candidates on a specific pathway, and 3) assessing the possibility of a PPI for a pair of proteins.

### Searching PPI candidates of a query protein

In this section, we describe the use of MEGADOCK-Web to search PPI candidates of a query protein. First, a user can search with a query word (for example, protein name, PDB ID, UniProt AC, etc.) typed in the “Search Single Protein” window in the Top page (Additional file 1: Figure S2), resulting in transition to the protein selection page (Additional file 1: Figure S3) showing the list of search results. In this page, protein structure hits with the query are listed accompanying identifiable protein information for each protein (PDB ID, chain, UniProt AC, protein name, and gene name). Clicking the “View” button takes users to the PPI prediction list page (Additional file 1: Figure S4). In the PPI prediction list page, proteins are shown in descending order of *PPIScore* and are paged per 10 items. The list contains information on the following three points for all the proteins in the database: 1) identification information, 2) *PPIScores* with the query protein, and 3) known PPIs for the query protein. Each row also has a “View” button to transit to the predicted complexes page (Additional file 1: Figure S5) between the query protein structure and the corresponding structure within the row.

On the predicted complexes page, 10 predicted complexes based on the results of the MEGADOCK dockings between the two proteins are displayed with 3D molecule viewers. From the “Download Complex in this page” link, 10 predicted complex structures can be downloaded as PDB files together in zip format. The table includes the following four columns: 1) rank (up to 1000) of the *DockingScore*, 2) *DockingScore*, 3) a molecule viewer displaying the 3D structure of the predicted complex, and 4) a PDB file download link for the complex structure. Molmil [45] is adopted as a molecular viewer because of its lightness and stability; the program enables users to rotate and enlarge the structure easily. It is also possible to download the docking results as a text file describing the translation distance, rotation angle of the ligand protein, and *DockingScore* (ZDOCK 3.0.1 and MEGADOCK output style).

### Searching PPI candidates on a specific pathway

Assuming that a user has a protein of interest known to affect a certain biochemical pathway with unknown interactions, this section describes how to discover PPI candidates in a specific pathway.

First, as in the previous section, the user can search a query by typing in the “Search Single Protein” window at the top of the page (Additional file 1: Figure S2) and select the protein structure in the protein selection page (Additional file 1: Figure S3). Next, in the PPI prediction list page (Additional file 1: Figure S4), the user can move to the pathway selection page (Additional file 1: Figure S6) by clicking the “Show Pathway” button. The pathway selection page displays a list of pathways containing at least one predicted binder. The predicted binder is defined as a protein with a *PPIScore* of greater than or equal to the user-defined threshold.

The threshold of *PPIScore* can vary from 6.0 to 12.0 with steps of 1.0 (default value is 8.0). Lowering the threshold increases the sensitivity and decreases the specificity. In the pathway selection page, links to the KEGG pathways to which the predicted binders belong are displayed. Note that the query protein does not always exist on the KEGG pathway; obtaining many predicted binders on the pathway without the query protein indicates a possibility of unknown interactions of the query protein in the pathway. Furthermore, in order to display only pathways with specific functions, it is possible to filter the pathways by inputting a word or phrase in the “Pathway Filtering” window at the top of the pathway selection page. Clicking the link takes the user to the corresponding KEGG page, in which the query protein is colored in blue, and predicted binders are in red.

### Assessing the possibility of PPIs for a pair of proteins

MEGADOCK-Web provides a functionality to assess the possibility of interactions with two proteins from functional and structural points of view. First, the user can search for the two proteins in the “Search Protein Pairs” window at the top of the page, resulting in transition to the protein pair selection page (Additional file 1: Figure S7). Selection of protein structures for each query is shown at the upper part of the page, whereas *PPIScore*s of all combinations of pairs of those protein structures are shown in descending order in the lower part of the page. The upper table has information similar to that of the protein selection page. If the structures of proteins to be used have already been specified, the predicted complexes page (Additional file 1: Figure S5) can be accessed by choosing the individual structure of each protein in the upper table.

The lower part of the page is useful if the structures of proteins to be used have not yet been determined. Since pairs of protein structures are presented in descending order of *PPIScores*, higher *PPIScore* structures (structures with higher PPI possibility) can be found easily. From the “View” link, it is also possible to transit to the predicted complex page (Additional file 1: Figure S5) of the corresponding proteins.

## Discussion

### How to utilize MEGADOCK-Web: Concrete examples

In this section, three case studies with specific proteins are explained to provide examples of the use of MEGADOCK-Web.

#### Case 1: APC and Axin (recalling a known PPI)

Cancer is one of the most serious diseases in humans. APC is a cancer-related protein; thus, PPI candidates with APC are likely to be related to cancer. In this section, we describe a search for PPI candidates of APC using MEGADOCK-Web.

**Fig. 4:**
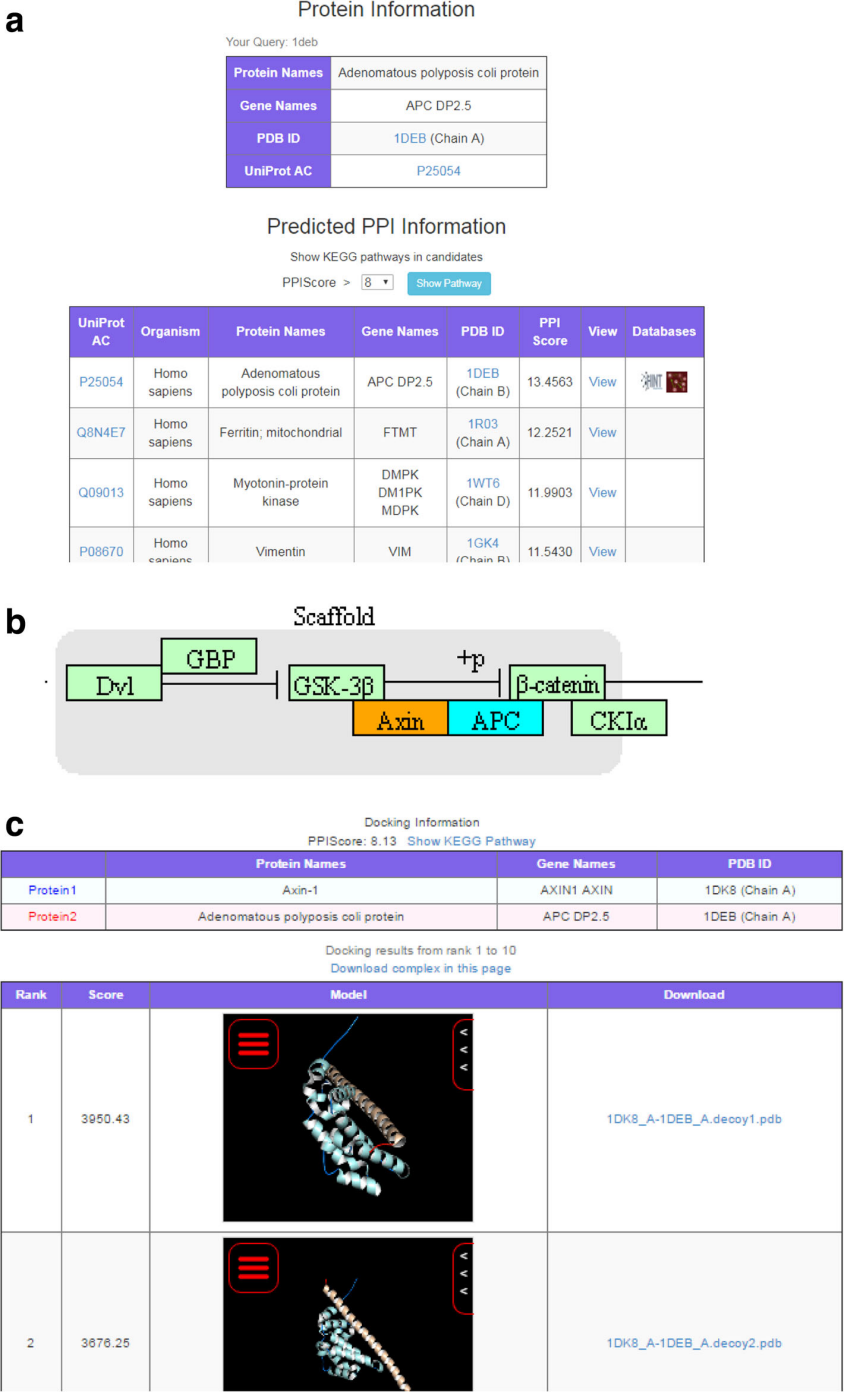
An example of page transition when searching for protein interaction candidates and APC. **a** The PPI prediction list page of APC. **b** Part of the KEGG pathway (breast cancer, hsa05224). The query protein (APC) is colored in blue, and the predicted binders (Axin) are in red. **c** The predicted complex page of 1DEB_A (APC) and 1DK8_A (Axin)

First, when APC is specified as a query, structure information of APC is displayed as search results. After selecting the structure of 1DEB_A (chain A of 1DEB) from several structures, users are taken to the PPI prediction list page (Fig. 4a). On this page, the pathway selection page with the threshold set to 8.0 is shown in order to view the KEGG pathways to which the predicted binders belong. Pathway filtering can be used to find cancer-related pathways. After filtering with the word “cancer”, the user can select breast cancer (KEGG ID: hsa05224). In the identified pathway, Axin (colored in red as a predicted binder) is present next to APC (colored in blue as the query protein; Fig. 4b). In order to more deeply assess the possibility of an interaction between APC and Axin, the user can use the pair query function and show the PPI prediction in detail. When searching APC and Axin as a pair query, some structures are found for both proteins. Selecting 1DEB_A and 1DK8_A results in transition to the predicted complex page (Fig. 4c). In the predicted complex page, predicted complexes are browsed, and the possible interactions can be assessed.

By following the above procedure, MEGADOCK-Web can facilitate the assessment of a specific PPI. In fact, the interaction between APC and Axin has been revealed experimentally [31].

#### Case 2: Sons of Sevenless (SOS) and SH2B adaptor protein 2 (SH2B2) (exploring a novel PPI)

The potential for identification of PPIs was described in the previous section. Here, we demonstrate the ability to identify a feasible new PPI using MEGADOCK-Web. We explain how to search unknown PPIs against SOS protein, which is also associated with cancer, as an example.

**Fig. 5:**
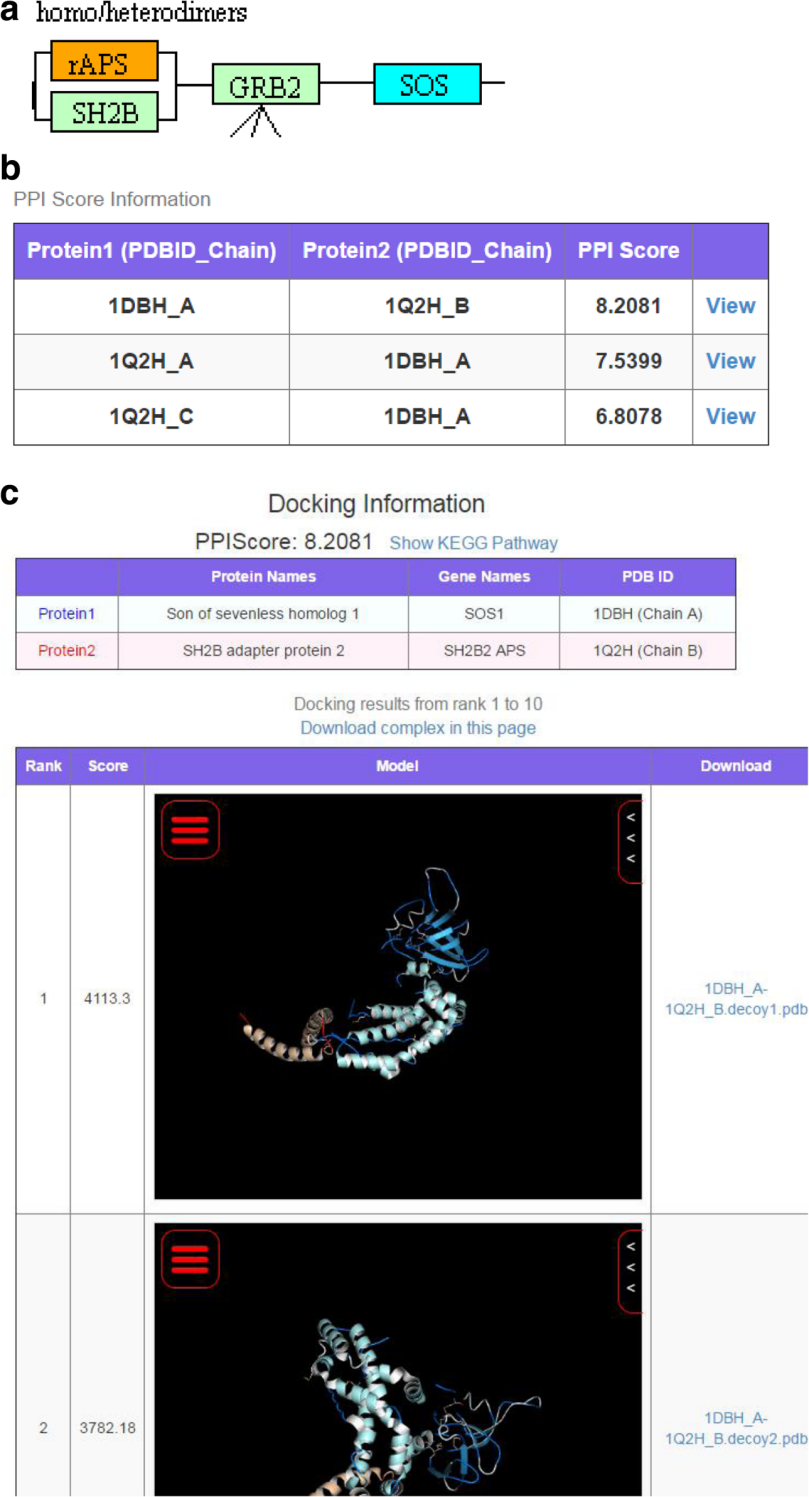
An example of page transition when searching for SOS protein interaction candidates. **a** Part of the KEGG pathway (neurotrophin signaling pathway, hsa04722). The query protein (SOS) is colored in blue, and the predicted binder (APS) is in red. Note that the notation of rAPS (rat ortholog of human APS) is considered to be a mistake in APS of KEGG. **b** The table in the lower part of the protein pair selection page for SOS and SH2B2 (APS). **c** The predicted complex page for 1DBH_A (SOS) and 1Q2H_B (SH2B2)

First, the pathway selection page is chosen, as described in the previous section, and the list includes the neurotrophin signaling pathway (hsa04722). In this pathway, an adaptor protein containing Pleckstrin homology (PH) and Src homology-2 (SH2) domains (APS), also known as SH2B2, is adjacent to SOS (Fig. 5a). Second, in order to search the possibility of an interaction between these proteins, searching SOS and SH2B2 as a pair query brings the user to the protein pair selection page, after which the user selects the predicted complex of 1DBH_A and 1Q2H_B with the highest score in the table (Fig. 5b). On the predicted complexes page (Fig. 5c), the possibility of an interaction is assessed. The *PPIScore* between 1DBH_A and 1Q2H_B was 8.2, indicating a possibility of a PPI.

The PPI database STRING DB stores experimental information and literature-based predictions [46]. Analysis using the database also supported the possibility of an interaction between SOS and SH2B2 when searching their UniProt ACs Q07889 and O14492, respectively. These results suggested that it may be useful to further investigate the interaction between SOS and SH2B2 (APS).

#### Case 3: Membrane Metallo-endopeptidase (MME) and methylenetetrahydrofolate dehydrogenase 1 (MTHFD1) (predicting the complex structure of a known PPI)

MME and MTHFD1 are a pair of proteins that interact in prostate cancer cells [47]; however, the complex structure of these proteins has not been determined. MEGADOCK-Web can provide predicted complex structures for these proteins.

**Fig. 6:**
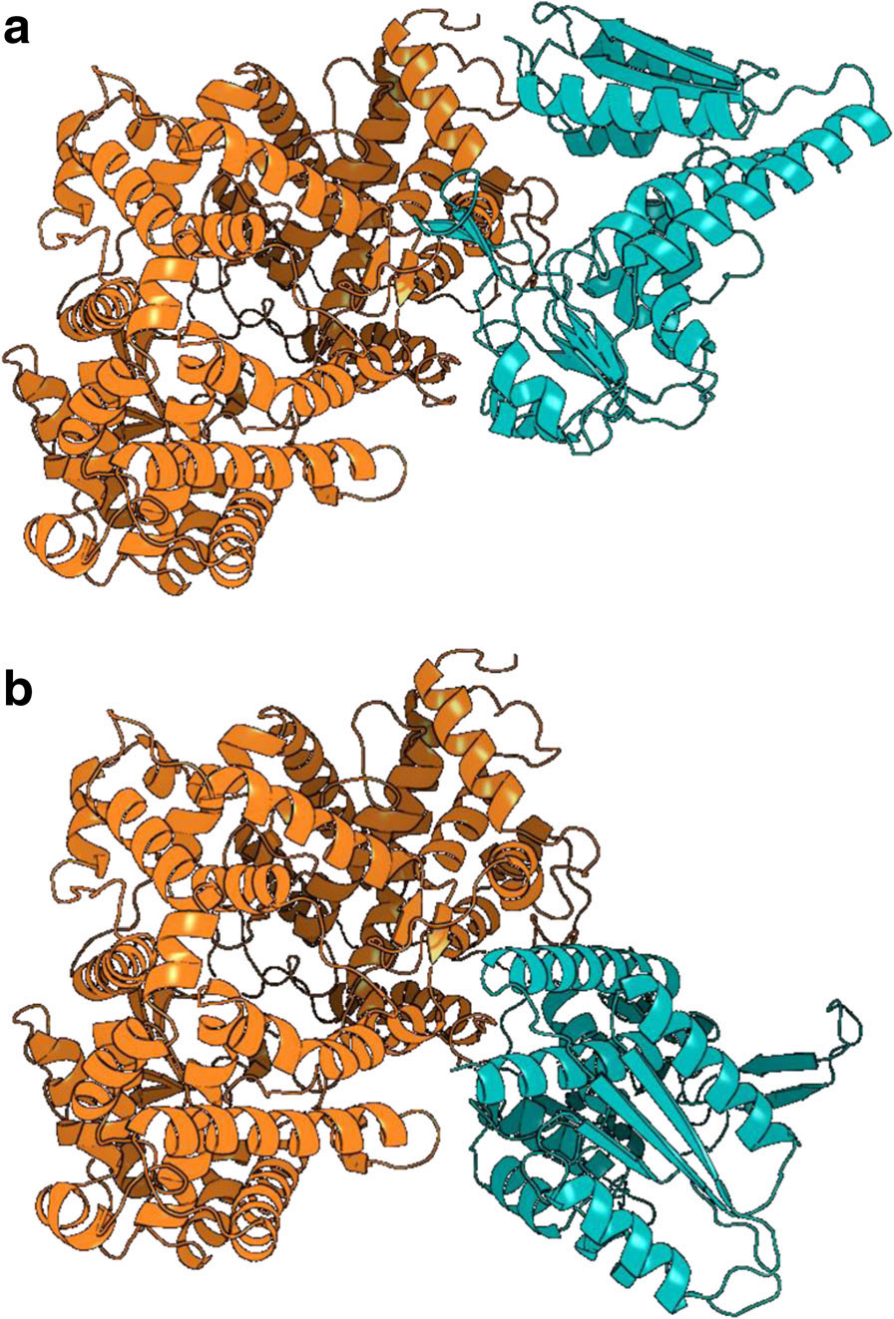
Predicted complex structures of 1R1H chain A and 1A4I chain B. **a** The predicted complexes for MME (1R1H_A) and MTHFD1 (1A4I_B), colored in orange and blue, respectively, with rank 1 protein-protein docking solutions; (**b**) predicted complexes with rank 2 protein-protein docking solutions. The difference in the position of MTHFD1 is RMSD = 43.7 Å

When MME and MTHFD1 are searched as queries, candidate structures are obtained. Here, 1R1H [48] describes the complex structure of MME and an inhibitor, and 1A4I [49] describes a homodimer complex structure of MTHFD1. Thus, the user may select 1R1H_A and 1A4I_B, and the predicted complexes are displayed in the predicted complex page. Figure 6a shows the predicted complex with Rank 1, and Fig. 6b shows that with Rank 2. The predicted complexes are created for different interaction surfaces. Therefore, users are able to discuss the possibility of PPIs on various interaction surface candidates.

### Comparison with other databases

Some existing databases contain 3D structural information similar to MEGADOCK-Web, such as PrePPI [16], HOMCOS [17], PRISM [28], and Interactome3D [29]. Table 1 shows a comparison of these databases and MEGADOCK-Web. MEGADOCK-Web was found to be superior in the following points: 1) provides many PPI predictions, 2) allows the user to search for pair proteins, 3) has a shorter response time, 4) permits viewing of some prediction complexes, and 5) allows mapping of proteins onto biochemical pathways.

**Table 1:** Comparison with other PPI prediction databases

	PrePPI [16]	HOMCOS [17]	PRISM [28]	Interactome3D [29]	MEGADOCK-Web
Number of PPI predictions	1,350,000	–	30,475	227,815	28,331,628
Comprehensive PPI prediction					✓
Search a single protein query	✓		✓	✓	✓
Search a pair protein query	✓	✓	✓	✓	✓
Viewing of multiple modeled complex structures		✓	✓	✓	✓
Mapping biochemical pathways				✓	✓
Template-free PPI prediction					✓

The numbers of archived PPI predictions as of June 1, 2017 are shown. Because HOMCOS is an on-demand prediction server, the number of PPI predictions is shown as ‘-’. ‘✓’ and blank cells indicate enable and disable

First, MEGADOCK-Web contains a larger number of PPI predictions than other databases because it uses MEGADOCK, a rapid rigid docking tool. For example, MEGADOCK-Web provides 10 times more PPI predictions than PrePPI and 100 times more PPI predictions than Interactome3D. Second, MEGADOCK-Web supports both single protein and paired protein queries. Thus, it is possible to conduct searches from various perspectives. Furthermore, since PrePPI displays only one prediction model, users cannot discuss the predicted complexes on various interaction surfaces. In contrast, MEGADOCK-Web enables users to investigate the complexes because up to 1000 candidate complexes can be shown in the predicted complexes page. Finally, since proteins are mapped on the biochemical pathways (KEGG pathways), MEGADOCK-Web helps users to discuss the possibility of PPIs based on the biological roles of the predicted interactions.

### Future plans

MEGADOCK-Web is expected to help to annotate proteins with unknown functions by providing comprehensive PPI predictions, irrespective of species. This program can be expected to help researchers discover new PPIs more broadly if we expand the database to include other species (e.g., *Saccharomyces cerevisiae*, *Escherichia coli* and *Mus musculus*). Although the current version of MEGADOCK-Web provides only human proteins, we will expand MEGADOCK-Web to include other species in the near future.

MEGADOCK-Web provides PPI predictions for proteins registered in the database. It will be useful to expand this system to perform docking calculations on-demand and allow browsing of PPI prediction results. With ordinary computers and implementations, long computation times are required for on-demand rigid body docking calculations; however, our system operates at multiple GPU computing nodes at high speeds. In addition, we have already completed a porting of MEGADOCK onto a public cloud computing resource (Microsoft Azure) that can use computing resources according to demand (Ohue M, et al: MEGADOCK-Azure: cloud-based high-performance protein-protein interaction prediction system on Azure HPC, in preperation). We will release a service for on-demand PPI prediction in the near future.

## Conclusions

In this paper, we described our new database, named MEGADOCK-Web, which is an integrated database of high-throughput structure-based PPI predictions. MEGADOCK-Web is freely available for use at http://www.bi.cs.titech.ac.jp/megadock-web/.

MEGADOCK-Web provides PPI predictions to users quickly by archiving exhaustive PPI predictions based on docking calculations. MEGADOCK-Web provides the following powerful functions: 1) searching PPI predictions by MEGADOCK, 2) providing annotations for each predicted protein pair with experimentally known PPIs, 3) visualizing candidate interactions with query proteins in biochemical pathways, and 4) visualizing predicted complex structures using a 3D molecular viewer. MEGADOCK-Web also gives comprehensive PPI prediction results in cooperation with biochemical pathways and enables users to easily and rapidly examine PPI possibilities. MEGADOCK-Web is expected to facilitate the discovery of new PPIs and protein functions.

## Availability and requirements

MEGADOCK-Web is freely available for use at http://www.bi.cs.titech.ac.jp/megadock-web/.

### Operating systems

MEGADOCK-Web is accessed from a web browser; therefore, it is platform-independent.

### Browsers

MEGADOCK-Web has been extensively tested with Google Chrome, Safari, Mozilla Firefox, and Microsoft Edge.

### Any restrictions for use

MEGADOCK-Web is free to use by everyone; there are no restrictions.

## Additional files

Additional file 1: **Supplementary Figures.** PDF file containing various figures with detailed MEGADOCK performance and MEGADOCK-Web screenshots. (PDF 819 kb)

## Abbreviations

3D: Three-dimensional
APC: Adenomatous Polyposis Coli
API: Application Programming Interface
APS: an adaptor molecule containing PH and SH2 domains
FFT: Fast Fourier Transform
GPU: Graphics Processing Unit
HTML: HyperText Markup Language
KEGG: Kyoto Encyclopedia of Genes and Genomes
MME: Membrane Metallo-endopeptidase
MTHFD1: Methylenetetrahydrofolate Dehydrogenase 1
PDB: Protein Data Bank
PPI: Protein-Protein Interaction
SH2: Src homology-2
SH2B2: SH2B adaptor protein 2
SOS: Son of Sevenless
