# Supplementary material for: MEGADOCK-Web: an integrated database of high-throughput structure-based protein-protein interaction predictions
Source: BMC Bioinformatics. 2018 May 8;19(Suppl 4):62. doi: 10.1186/s12859-018-2073-x (PMC5998897; doi:10.1186/s12859-018-2073-x)
Supplement: Supplementary file 1 — Supplementary Figures. PDF file containing various figures with detailed MEGADOCK performance and MEGADOCK-Web screenshots. (PDF 819 kb) [file 12859_2018_2073_MOESM1_ESM.pdf]

Additional File

MEGADOCK-Web: an integrated database of high-throughput structure-based protein-protein interaction predictions

Takanori Hayashi[1,2], Yuri Matsuzaki[2], Keisuke Yanagisawa[1,2], Masahito Ohue[1,3,4*], Yutaka Akiyama[1,2,3,4,5*]

[1] Department of Computer Science, School of Computing, Tokyo Institute of Technology, 2-12-1 W8-76 Ookayama, Meguro-ku, Tokyo 152-8550, Japan

[2] Education Academy of Computational Life Sciences (ACLS), Tokyo Institute of Technology, 2-12-1 W8-93 Ookayama, Meguro-ku, Tokyo 152-8550, Japan

[3] Advanced Computational Drug Discovery Unit (ACDD), Institute of Innovative Research, Tokyo Institute of Technology, 4259 Nagatsutacho, Midori-ku, Yokohama, Kanagawa 226-8501, Japan

[4] AIST-Tokyo Tech Real World Big-Data Computation Open Innovation Laboratory (RWBC-OIL), National Institute of Advanced Industrial Science and Technology (AIST), 1-1-1 Umezono, Tsukuba, Ibaraki 305-8560, Japan

[5] Molecular Profiling Research Center for Drug Discovery (molprof), National Institute of Advanced Industrial Science and Technology (AIST), 2-4-7 Aomi, Koto-ku, Tokyo 135-0064, Japan

[*]Corresponding authors

Email addresses:

TH: hayashi@bi.c.titech.ac.jp
YM: matsuzaki@acls.titech.ac.jp
KY: yanagisawa@bi.c.titech.ac.jp
MO: ohue@c.titech.ac.jp
YA: akiyama@c.titech.ac.jp

Supporting Figures

Figure S1: Precision and recall at each threshold value with complete cross-docking on protein-protein docking benchmark version 4.0*

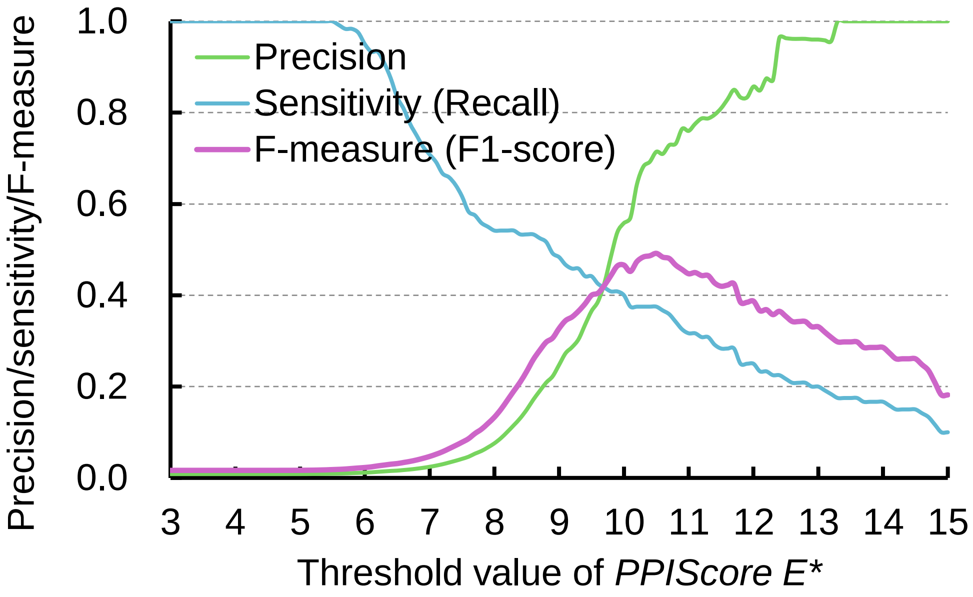

*Hwang H, Vreven T, Janin J, Weng Z. Protein-protein docking benchmark version 4.0. Proteins 2010;78:3111-4.

Figure S2: MEGADOCK-Web top page

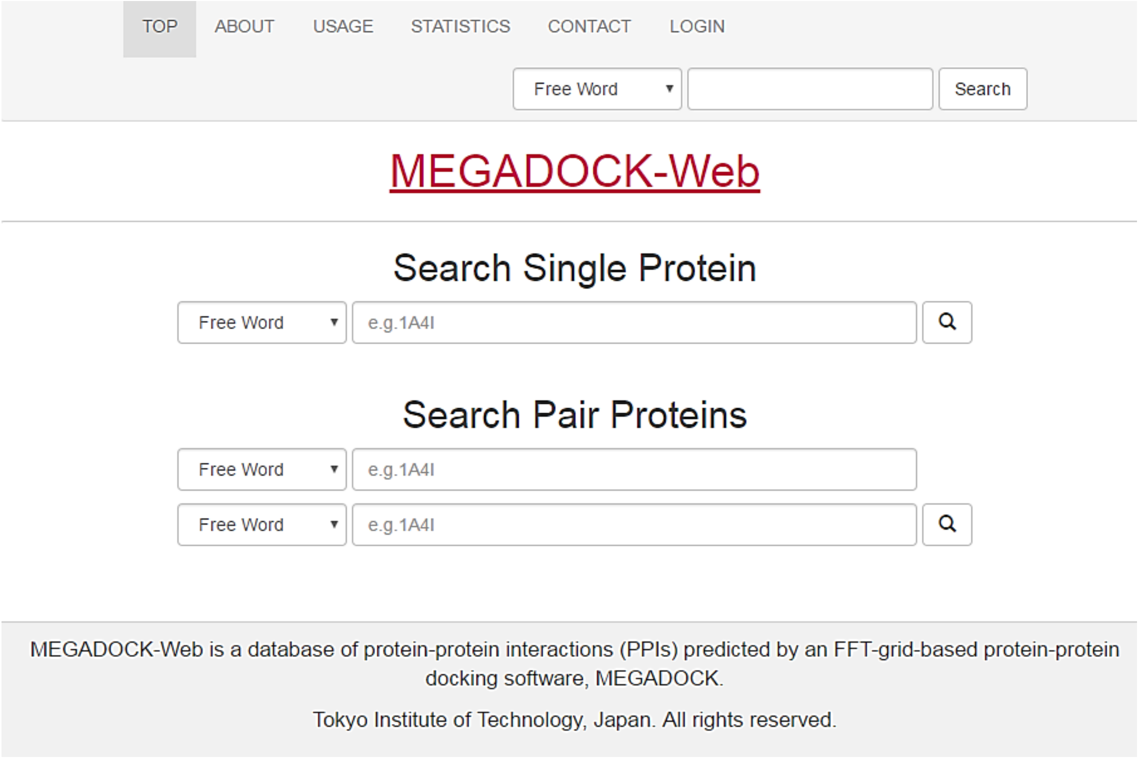

In the top page, there are two functions, i.e., "Search Single Protein" and "Search Pair Proteins".

Figure S3: Protein selection page

There are 3 matches for the query "1a12". Please click "View" to go to your protein page.

UniProt AC	Organism	Protein Names	Gene Names	PDB ID	Chain	
P18754	Homo sapiens	Regulator of chromosome condensation	RCC1 CHC1	1A12	A	View
P18754	Homo sapiens	Regulator of chromosome condensation	RCC1 CHC1	1A12	B	View
P18754	Homo sapiens	Regulator of chromosome condensation	RCC1 CHC1	1A12	C	View

« 1 »

This page shows candidate protein structure hits after making a query. The table contains basic information on proteins, including PDB ID, chain, UniProt AC, gene name, and protein name for each protein.

Figure S4: *PPIScore* list page

Protein Information

Your Query: 1oa8

Protein Names	Ataxin-1
Gene Names	ATXN1 ATX1 SCA1
PDB ID	1OA8 (Chain A)
UniProt AC	P54253

Predicted PPI Information

Show KEGG pathways in candidates

PPIScore > 8 ▼ Show Pathway

UniProt AC	Organism	Protein Names	Gene Names	PDB ID	PPI Score	View	Databases
P54253	Homo sapiens	Ataxin-1	ATXN1 ATX1 SCA1	1OA8 (Chain B)	20.3817	View	
P54253	Homo sapiens	Ataxin-1	ATXN1 ATX1 SCA1	1OA8 (Chain D)	18.2159	View	
P21583	Homo sapiens	Kit ligand [Cleaved into: Soluble KIT ligand]	KITLG MGF SCF	1EXZ (Chain C)	10.8461	View	
P01130	Homo sapiens	Low-density lipoprotein receptor	LDLR	1IJQ (Chain B)	10.5583	View	
Q15797	Homo sapiens	Mothers against decapentaplegic homolog 1	SMAD1 BSP1 MADH1 MADR1	1KHU (Chain A)	10.4384	View	
O14936	Homo sapiens	Peripheral plasma membrane protein CASK	CASK LIN2	1KWA (Chain B)	10.3444	View	
P23381	Homo sapiens	Tryptophan--tRNA ligase; cytoplasmic [Cleaved into: T1-TrpRS; T2-TrpRS]	WARS IFI53 WRS	1ULH (Chain A)	9.9297	View	
P04234	Homo sapiens	T-cell surface glycoprotein CD3 delta chain	CD3D T3D	1XIW (Chain B)	9.9288	View	
Q9HAN9	Homo sapiens	Nicotinamide/nicotinic acid mononucleotide adenylyltransferase 1	NMNAT1 NMNAT	1KQN (Chain B)	9.9102	View	
Q9UKK6	Homo sapiens	NTF2-related export protein 1	NXT1	1JKG (Chain A)	9.9045	View	

« 1 2 3 »

This page shows candidate proteins interacting with the query protein on the basis of *PPIScore*. Specifying the threshold of *PPIScore* and pressing the “Show Pathway” button at the top of the page results in transition to the pathway selection page, whereas clicking the “View” link in the table takes the viewer to the predicted complex page.

Figure S5: Predicted complex page

Docking Information

PPIScore: 11.1101 Show KEGG Pathway

	Protein Names	Gene Names	PDB ID
Protein1	N--L-asparaginase [Cleaved into: Glycosylasparaginase alpha chain; Glycosylasparaginase beta chain]	AGA	1APY (Chain A)
Protein2	Regulator of chromosome condensation	RCC1 CHC1	1A12 (Chain A)

Docking results from rank 1 to 10

Download complex in this page

Rank	Score	Model	Download
1	6314.28	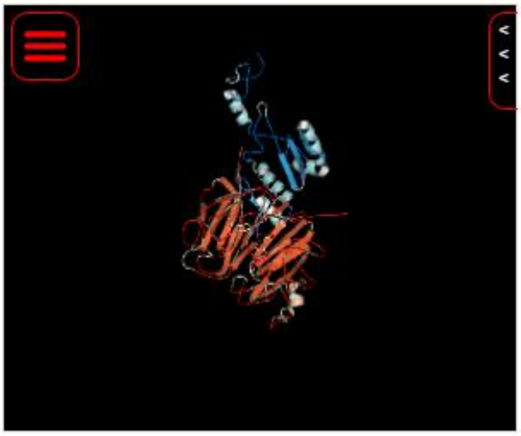	1APY_A-1A12_A.decoy1.pdb
2	6142.25	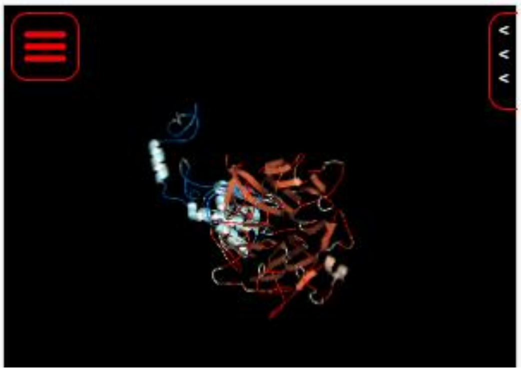	1APY_A-1A12_A.decoy2.pdb

In this page, complex structures predicted by MEGADOCK for the two proteins are displayed with the 3D molecular viewer, Molmil. A PDB-format file can be downloaded for each predicted complex, and 10 PDB files can be compressed into zip format from the "Download complexes in this page" link at the top of the page.

Figure S6: Pathway selection page

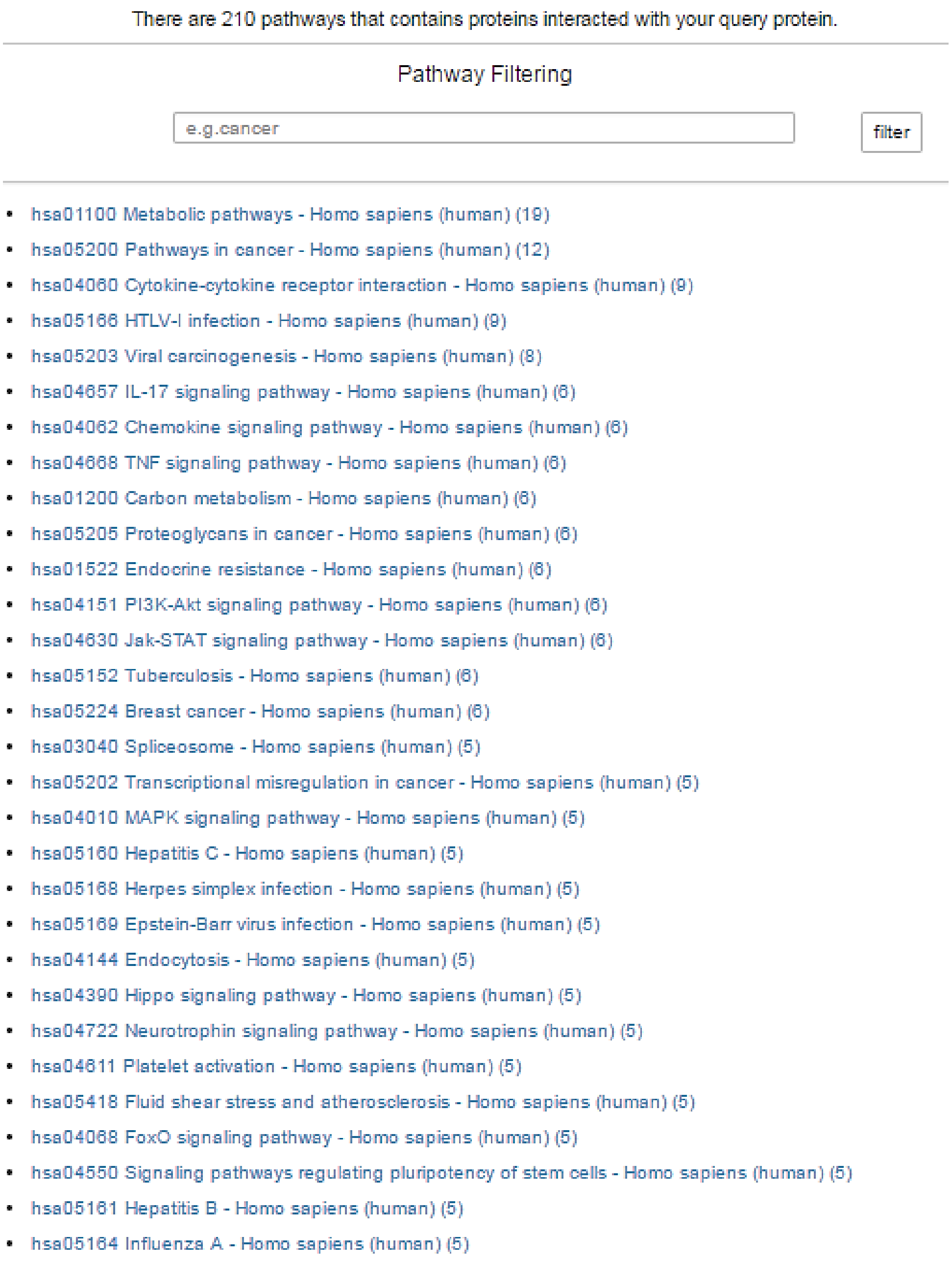

In this page, the pathways to which the predicted binders belong are shown as a list. The pathways can be filtered according to a word or phrase typed in the "Pathway Filtering" box at the top of the page. The number in parentheses next to the pathway name indicates the number of predicted binders included in the pathway.

Figure S7: Protein pair selection page

Your Query 1:axin

	UniProt AC	Organism	Protein Names	Gene Names	PDB ID	Chain
○	O15169	Homo sapiens	Axin-1	AXIN1 AXIN	1DK8	A
○	O15169	Homo sapiens	Axin-1	AXIN1 AXIN	1EMU	A
○	P54253	Homo sapiens	Ataxin-1	ATXN1 ATX1 SCA1	1OA8	A
○	P54253	Homo sapiens	Ataxin-1	ATXN1 ATX1 SCA1	1OA8	B
○	P54253	Homo sapiens	Ataxin-1	ATXN1 ATX1 SCA1	1OA8	C
○	P54253	Homo sapiens	Ataxin-1	ATXN1 ATX1 SCA1	1OA8	D

Your Query 2:apc

	UniProt AC	Organism	Protein Names	Gene Names	PDB ID	Chain
○	P25054	Homo sapiens	Adenomatous polyposis coli protein	APC DP2.5	1DEB	A
○	P25054	Homo sapiens	Adenomatous polyposis coli protein	APC DP2.5	1DEB	B
○	P25054	Homo sapiens	Adenomatous polyposis coli protein	APC DP2.5	1M5I	A

Submit

PPI Score Information

Protein1 (PDBID_Chain)	Protein2 (PDBID_Chain)	PPI Score	
1DEB_B	1OA8_D	8.1506	View
1DK8_A	1DEB_A	8.13	View
1DEB_B	1OA8_C	7.8709	View
1OA8_D	1DEB_A	7.6317	View
1OA8_A	1DEB_B	7.1811	View
1OA8_A	1DEB_A	7.0214	View
1EMU_A	1M5I_A	6.9516	View
1M5I_A	1OA8_D	6.8361	View
1EMU_A	1DEB_A	6.836	View
1M5I_A	1OA8_C	6.7529	View
1DK8_A	1M5I_A	6.7018	View
1DEB_B	1DK8_A	6.5972	View
1DEB_B	1OA8_B	6.55	View

In this page, a list of candidate hits for the two queries is displayed. In addition, at the bottom of the page, a table showing the all-to-all *PPIScore* of the protein hit by query 1 and the protein hit by query 2 is displayed.
